# The Educational Situation Quality Model: A New Tool to Explain and Improve Academic Achievement and Course Satisfaction

**DOI:** 10.3389/fpsyg.2019.01692

**Published:** 2019-07-18

**Authors:** Fernando Doménech-Betoret, Amparo Gómez-Artiga, Laura Abellán-Roselló

**Affiliations:** ^1^Developmental and Educational Psychology, Jaume I University, Castellón de la Plana, Spain; ^2^Developmental and Educational Psychology, University of Valencia, Valencia, Spain

**Keywords:** academic achievement, course satisfaction, educational model, research in the classroom, teacher training, learning outcomes

## Abstract

Students’ academic achievement is a major concern among countries. Governments spent a lot of money on education to improve students’ competences at all levels of education. Despite the enormous amount of money invested and the reforms made to curricula in many countries in recent years, these measures are not generally producing the desired results according to the data of International Performance Measurement programs for students (e.g., Program for International Student Assessment-PISA by OECD). Given the importance of this issue, this article presents an instructional-motivational model developed in the last decade to explain and improve students’ learning outcomes, e.g., academic achievement and course satisfaction, entitled the “The Educational Situation Quality Model” (MOCSE, acronym in Spanish). Unlike other educational models, MOCSE offers an integrative teaching-learning approach to explain learning outcomes. By taking the educational setting as a unit of analysis, this proposal introduces a new perspective into the existing literature to predict students’ achievement and course satisfaction by combining contributions from relevant psycho-educational theories, such as: “The Job Demands-Resources Model,” “The Expectancy-Value Theory,” and “The Achievement Goal Theory.” Besides being a conceptual framework to guide research, it also provides a methodological way to improve teacher practice and learning outcomes. In this article we first briefly explain the main model’s characteristics and functioning from the student perspective and, second, based on the MOCSE, we offer some keys for teachers to improve academic achievement and students’ course satisfaction for a specific curricular subject. Finally, future proposals and challenges are discussed. Questionnaires are provided in the Annex.

## Introduction

Students’ learning outcomes, such as academic achievement, is a major concern for teachers and governments, and one of the most important issues in the field of education and educational psychology, as proven by the large amount of research in the existing literature that focuses on this topic.

However, most of the research conducted to explain or predict learning outcomes which we can find in the literature, have two important limitations. First, no consensus theoretical model is used as a basis. Each author centers on specific variables according to his/her theoretical tradition, which makes comparing and interpreting the results difficult. Second, many of the models used as a reference to guide research in the classroom lack a scientific and solid theoretical basis by, for instance, providing a conceptual framework to consider the teaching and the learning process independently. These limitations hinder progress from being made in this field.

“The Educational Situation Quality Model” (MOCSE, acronym in Spanish), devised by Doménech-Betoret (2006, 2013, 2018), which we present herein, attempts to overcome the aforementioned shortcomings by providing an integrated and scientific approach to explain students’ learning outcomes, such as academic achievement and course satisfaction. Unlike other existing ES models in the literature that lack a solid theoretical basis, MOCSE has been configured by combining contributions from relevant psycho-educational theories, such as: “The Job Demands-Resources Model,” “The Expectancy-Value Theory,” and “The Achievement Goal Theory.” Moreover, previous research that has focused on MOCSE has allowed the original proposal to be refined and has improved the model’s predictive capacity. Finally, the model besides being a conceptual framework to guide research, it also provides a methodological way to improve teacher practice and learning outcomes.

As the studies conducted to date have been done exclusively with students (and not with teachers) from University and Secondary Education levels, this is why we present the structural configuration of the model centered on student in the current study. Although more research is needed, the results obtained to date seem to support the MOCSE model’s viability with students. In the current study we explain the model’s characteristics, how it works and its use to improve learning. Investigating the MOCSE model centered on the teacher is a future challenge.

## Theoretical Framework: The Educational Situation Quality Model Focused on Students

**FIGURE 1:**
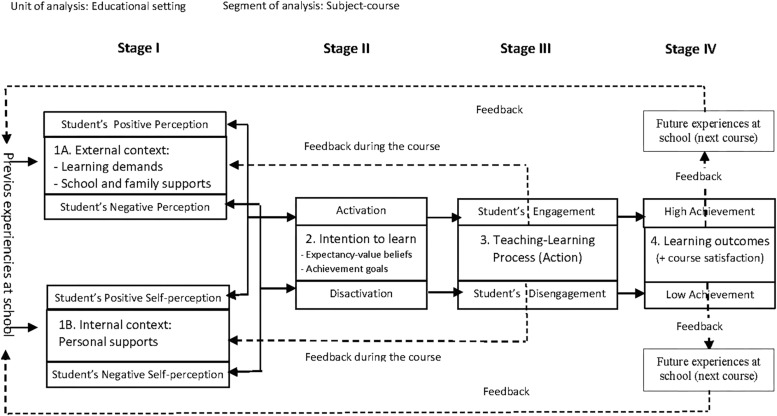
MOCSE focused on students: structural configuration and relationship between variables.

What do a student being more engaged than others, learning more than others and obtaining better academic results depend on? What can I, as a teacher, do so that students engage more, learn and, consequently, obtain better results in a given subject? Answering both questions is crucial to implement actions and programs to improve learning outcomes. This article aims to shed light on the answers to both questions from a new approach provided by the Educational Situation Quality Model (Doménech-Betoret, 2006, 2013, 2018). The studies conducted to date about MOCSE have been done from only the student perspective. The data obtained seem to endorse the configuration of the model centered on the student (Doménech-Betoret, 2006; Doménech-Betoret et al., 2014, 2019; Abellán-Roselló, 2016). The structural configuration of model centered on students is displayed in Figure 1.

As we can see in Figure 1, students’ perception of learning demands, and the perceptions of the supports they are provided with to overcome such demands (Stage I: Appraisal stage), predict intention to learn (Stage II: Intention activation) which, in turn, affect the level of engagement (behavioral, cognitive, relational, and affective) adopted by students during the teaching-learning process (Stage III: Teaching-Learning process) which, in turn, finally has an effect on learning outcomes, such as academic achievement and course satisfaction (Stage IV: Learning outcomes). The whole model pivots around the intention to learn, where the components from Stage I are considered antecedents or predictive variables, whereas those from Stages III and IV are considered consequences or outcome variables. As the course unfolds, students receive continuous feedback about their progress that affects their perception of the demands required and supports received. Consequently, students’ perceptions are continuously updated and changing. The model operates as a system, insofar that the changes in one of its five components affect all the others.

This proposal integrates three important motivational theories to explain students’ involvement (engagement) and learning outcomes: Job Demands and Resources Model (Demerouti et al., 2001; Bakker and Demerouti, 2007), Expectancy-Value theory (Eccles and Wigfield, 2002), and Achievement goal theory (Dweck and Leggett, 1988; Nicholls, 1989; Ames, 1992; Wigfield and Eccles, 2000).

### Stage I: Demands and Supports for Students in the Classroom Context

Applying this theory to the school context, first, requires a thorough analysis of what the specific demands required of students and teachers are and, second, which resources/supports are provided to them (students and teachers) to complete those demands. We assume, as a general principle, that students’ and teachers’ perceptions of the learning and teaching demands, respectively, and the resources/supports they are provided with (or expected to be provided with until the end of the course) in order to overcome those demands, play an important role in the quality of the teaching-learning process (T-L) undertaken, and determine, to a large extent, academic achievement and course satisfaction.

Centered on the students’ learning process, the JD-R (Demerouti et al., 2001; Bakker and Demerouti, 2007) was used to study students’ perceptions of a specific educational setting in terms of learning demands and the resources/supports (external and internal) they need to overcome such demands. The selected demands or tasks that students have to meet to pass a specific subject matter (e.g., problem solving, assignments, tasks, oral presentations, lab work, etc.) are included and described in the subject’s planning. They are all subordinated and aim to fulfill learning objectives (the most important demands). Students may acquire information about their required learning demands at the beginning of the course in the subject syllabus or when the teacher introduces the subject’s planning.

We begin with a basic premise (Doménech-Betoret et al., 2014; Abellán-Roselló, 2016; Doménech-Betoret, 2018), the perception that each student forms of; first, the scheduled learning demands they must complete to pass a given subject; second, the support that they receive or perceive they will have mainly from the teacher and family to face these demands is crucial information to activate students’ intention to learn. Consequently, they will decide to engage, or not, in learning a given subject.

#### Demands of Students

The learning demands in the context of a specific subject, refer basically to the tasks that students have to complete and the contents they have to study to achieve the programmed objectives and pass the subject.

Two major features can be distinguished from a learning demand: (1) the typology, related to the competence that the teacher wishes to be developed (cognitive, socio-affective, etc.); (2) the motivational characteristics of demand, which enable students’ intention to learn activation. The motivational characteristics of demands (specifically, activities and tasks that students are required to complete to overcome a specific subject) should meet five major requirements to activate students’ intentions to learn and encourage students to engage in the teaching learning process (Doménech-Betoret and Abellán-Roselló, 2017): (a) connect with students’ living environment; (b) connect with students’ interests and needs; (c) connect with what students already know; that is, with their previous knowledge (see Ausubel); (d) have a moderate level of difficulty (neither too easy, nor too difficult); in other words, the level of difficulty should be located, according to Vygotsky, in the zone of proximal development (ZPD); (e) be useful for students at a personal or a professional level; (f) make sense in the context of the subject matter. The more requirements a demand has, the more motivating it will be.

#### External and Internal Support Resources to Overcome Required Demands

Regarding the external resources/supports provided by teachers, “most authors have usually distinguished between affective/emotional or instructional/instrumental supports, but there is lack of consistency in the terminology used” (Doménech-Betoret, 2018). Instructional support provided by teachers aims to facilitate students’ content domain which, in turn, will contribute to achieve learning demands. The affective support provided by teachers aims to meet students’ psychological needs and wishes in the classroom context. It will contribute to activate positive emotions and to generate a healthy classroom climate. Students’ course satisfaction is related to the emotions experienced by students during the course.

Regarding the external resources/supports provided by the family, previous studies have found that when parents or family members provide academic (e.g., assisting with homework) or affective (e.g., recognizing effort) support, students’ academic achievement improves (Jelas et al., 2016). Research has also found that parents’ support and involvement positively influence children’s perception of their own abilities and also how they value the subjects taught at school (Rodríguez et al., 2017).

Finally, regarding internal support, students’ self-beliefs related to their personal identity and self-competence (e.g., self-esteem, self-efficacy, self-concept, self-control, self-confidence, etc.) are considered important internal resources/support to predict motivation and goal setting (Doménech-Betoret, 2006; Doménech-Betoret et al., 2014, 2017; Abellán-Roselló, 2016). Students’ positive self-beliefs act as personal-internal support resources that may shape their initial perception of the T-L process in terms of the demands and supports provided to achieve the planned learning objectives (Doménech-Betoret, 2018).

The teaching supports considered to date in the research carried out in the MOCSE context are listed below in Table 1. Bearing in mind that three fundamental levels/dimensions exist in an ES situation, namely Academic, Interpersonal and Intrapersonal, which need to be dealt with, we classified teaching supports according to the importance or effect that each specific support has on all three levels.

**TABLE 1:** Classification of the teacher supports considered to date in the research conducted in the MOCSE context.

**TEACHER SUPPORTS considered to date in the MOCSE context**				
**Teacher support**	**(1) Acad.**	**(2) Inter.**	**(3) Intra.**	**Item example**
(1) Content comprehension support	^*^			“The teacher’s explanations are clear and understandable”.
(2) Motivational support			^*^	“From the beginning, the teacher made an effort to arouse our curiosity and interest in this subject”.
(3) Formative evaluation (teacher feedback)	^*^			“The evaluation system attaches much importance to students’ continued work and the teacher’s feedback”.
(4) Relational support		^*^		“The teacher comes over as being willing and open to dialogue”.
(5) Competence support			^*^	“From the beginning, the teacher has conveyed to us the idea that we are all qualified to pass this subject if we propose to do so”.
(6) Recognition support			^*^	“When we do things right, this teacher values it and praises us for it”.
(7) Assisting students to improve achievement (study guidance)	^*^			“The teacher has guided us how to learn more and be better in this subject”.
(8) Autonomy support			^*^	“The teacher gives us a chance to focus and organize the work of the topics as we wish”.
(9) Providing didactic resources to study support	^*^			“The teacher has provided us with enough varied materials to study and work on this subject”.
(10) Teacher’s accessibility		^*^		“This teacher quickly and effectively answers the questions raised by students”.

^*^Indicates the classroom level affected by such support. (1) Acad., academic level of the educational setting; (2) Inter., interpersonal level of the educational setting; (3) Intra., intrapersonal level of the educational setting.

(a) Interpersonal level: it refers to personal relationships (teacher-students, students-students). The teacher’s obligation in this area is to improve the classroom climate by managing these interpersonal relationships. Empathy and respect in dealings between teacher-students and among peers are two fundamental requirements to achieve a good classroom climate.
(b) Intrapersonal level: it refers to the relationship with oneself. Having low self-esteem, low self-concept, undervalued perception of self, etc., generates fears and insecurities (fear of failure, humiliation, etc.) in students, especially adolescents. These fears hinder learning because these students pay more attention to protect themselves than to progress and master the subject.
(c) Academic level: it is a consequence of the two previous levels. It refers to the teaching and learning of curricular content. Successful teachers cover all three levels, but some teachers only cover this level and ignore the above-mentioned two.

The decision to set a goal intention (i.e., choosing a desired end state to strive for) is commonly assumed to depend on both the desirability and the feasibility of a certain outcome (e.g., Fishbein and Ajzen, 2010). Goals are most likely to be set when the anticipated end state is subjectively evaluated as both desirable (I want X!) and feasible (I am confident that I can achieve X!). Thus, from a psychological perspective, a strong desire to attain a goal is not sufficient for the formation of a goal intention; in addition, one must be confident that the chances of attaining the goal are high.

### Stage II: Intention to Learn Measured Through Expectancy-Value Beliefs and Achievement Goals

Intentionality is considered the immediate previous step of action. According to the theory of Action-Control (Heckhausen and Kuhl, 1985), intention to learn is a motivational state that is generated in the subject before initiating behavior to achieve a certain goal, and is associated with decision making. The decision to set a goal to strive for is usually assumed to depend on both the desirability and the feasibility of reaching that goal (Fishbein and Ajzen, 2010). In other words, a certain goal is more likely to be set when individuals feel a strong desire to attain it and when they are confident that they can achieve it. The formation of a goal intention is governed by motivational principles.

According to a basic educational rule accepted by the majority of authors, successful learning requires “students’ intention to learn and the teacher’s intention to teach to be activated at the beginning of the educational process, and have to remain active until the process ends” (Doménech-Betoret, 2018). Intention to learn is activated on the first days of the course, and basically depend on students’ perception of both the required demands and the supports provided by the teacher. However, it is assumed that this may change and fluctuate during the T-L process as a result of constant (re)appraisals made by students of the support provided to fulfill learning demands.

#### The Expectancy-Value Theory

The expectancy-value theory (see Eccles and Wigfield, 2002 for a modern version of this theory) is grounded in the social cognitive view of motivation. In this tradition, psychologists claim that individuals’ choice, persistence and vigor invested in performance can be basically predicted and explained by their beliefs about how well they will do in the task and the value that the task has for them (Atkinson, 1957; Wigfield and Eccles, 1992; Wigfield, 1994). The three major constructs that are considered important for psychologists in this tradition are listed below:

(a) Expectancy for success (Will I succeed in this subject?). This construct is defined as “individuals’ beliefs about how well they will do in upcoming tasks” Eccles and Wigfield (2002, p. 119). Expectancy for success is more future-oriented than simple self-perceptions of competence, and it refers to students’ actual beliefs about their future expectancy for success. Expectancy for success usually comprises outcome expectancy and self-efficacy expectancy (Liem et al., 2008). Both terms were introduced by Bandura (1986), who differentiated between “self-efficacy or efficacy expectations” and “outcome expectancy.” This author defined the former as an individual’s belief in his/her own capability to accomplish a given task, while the latter is considered a person’s belief that the effort he/she invests will lead to the desired outcome (Bandura, 1986).
(b) Expectancy for enjoyment (How will I feel studying this subject?). Given the importance of students’ affective state for their engagement while learning, this affective component has been considered by expectancy-value theorists to be crucial (Pintrich, 1989; Pintrich and De Groot, 1990). It refers to the feelings that students expect to experience during the course, which derive from the teacher-students, content-students and peer relationships.
(c) Task/subject value (What value does this subject have for me?). Task value refers to students’ beliefs about if a task or subject is worth pursuing. According to Liem et al. (2008), students’ beliefs about if a task or subject is worth pursuing is a key component for understanding students’ behaviors and learning outcomes. The term “value” seems a simple construct, but it is not because it has different understandings. For instance, an object can have an intrinsic, extrinsic and instrumental value. The modern expectancy-value theory (Eccles and Wigfield, 2002; Eccles, 2009) distinguishes four task-value components that we applied to a course subject to assess the subject matter value: utility, importance, interestingness and cost.
  Finally, given the importance of the attributional theory in students’ motivation, an additional construct was considered and added to the three aforementioned ones.
(d) Expectancy of control (To what extent does it depend on me to pass or fail this subject?). Given the importance of the attributional theory (Rotter, 1966; Weiner, 1992) in students’ motivation, the construct of control was taken into account in the way of expectations, and an additional fourth construct, called “expectancy of control,” was added to the three above- mentioned ones. The theorists of this tradition stress the idea that causal interpretations or attributions made by students of academic results (successes and failures) determine their motivation and efforts to a great extent. For instance, student motivation will suffer, and students will most certainly not make much effort to study a subject, if they consider that it does not depend on them (no matter how much effort they make) to pass or fail it, but on other factors beyond their control; e.g., if they get on well with the teacher, the teacher’s mood when correcting exams, luck, etc. Accordingly, if the attributions that students make of their academic successes and failures are controllable, they will be more motivated to learn than if their causal attributions are uncontrollable.

#### The Achievement Goal Theory

The achievement goal theory (Dweck and Leggett, 1988; Nicholls, 1989; Ames, 1992; Wigfield and Eccles, 2000) argues that “the purposes that students hold for engaging in a specific learning task or in a learning process followed with a specific subject matter (i.e., their achievement goals) are an important antecedent to their achievement-related processes and outcomes” (Liem et al., 2008, p. 487). Three main goals are usually considered by researchers in this field: mastery goals, performance goals and performance-avoidance goals. The students who adopt mastery goals focus on developing one’s competence to achieve a task or to pass a subject. The students who set a performance goal are concerned about others demonstrating their competence. Finally, the students who set a performance-avoidance goal wish to avoid social judgments and humiliation by others, such as the teacher or peers. For more in-depth details, see the study carried out by King and Mclnerney (2014). Previous research has found associations between the achievement goals adopted by students and outcomes variables, such as engagement/disengagement (Harackiewicz et al., 2000; Liem et al., 2008); academic achievement (Pintrich, 2000; Roebken, 2007; Diseth et al., 2012; Wei-Wen and Yi-Lee, 2015); and student satisfaction and enjoying class (Harackiewicz et al., 2002; Roebken, 2007).

In conclusion, we consider expectancy-value beliefs and achievement goals the two main dimensions to assess “intention to learn” (for more details, see Doménech-Betoret, 2018). Prior research (Plante et al., 2013) seems to indicate that achievement goals are well explained by expectancy-value constructs, but not the other way around. That is why we have placed expectancy-value constructs as the first dimension and achievement goals as the second dimension. A high score in both dimension indicates high intention to learn, whereas a low score in both dimension indicates low intention to learn.

### Stage III: The Teaching-Learning Process: Students’ Engagement

Students’ engagement is crucial for academic outcomes and school success, that’s why engagement is one of the most important issues of educational research. A review of the literature reveals that no consensus has been reached by authors about defining this construct (Schaufeli, 2013) and, consequently, about how it should be measured. Broadly speaking, in the school context, it is generally assumed that engagement occurs when students are involved in learning tasks, and is characterized by students’ continuous effort, determination and perseverance in learning (Liem et al., 2008). On the contrary, disengaged students are characterized by lack of interest, inaction and the use of avoidance strategies. Avoidance strategies are considered a negative indicator of students’ engagement. Students use avoidance strategies when they give up, quit or disengage in their learning tasks related to a specific subject matter. In short, we can roughly state that *engagement* refers to involvement or participation; conversely, non-engagement, or disengagement, refers to withdrawal or apathy.

In recent years, students’ engagement has been viewed as a multidimensional concept (Schaufeli, 2013). Centered on an educational setting, engagement is usually examined by considering how students behave (behavioral engagement), feel (affective or emotional engagement), think (cognitive engagement), and socialize or interact (social or relational engagement) in the classroom. Behavioral engagement is more observable and easily measurable. It usually includes actions and efforts made by students (Fredricks et al., 2004; Handelsman et al., 2005), such as, asking questions, taking an active part in class, paying attention and taking notes, participating in learning activities, etc. Cognitive engagement refers to how students feel about themselves and how effective the processing strategies or skills they use to master certain tasks are (Metallidou and Vlachou, 2007), such as, synthesizing information, highlighting the main ideas, etc. Emotional engagement has to do with the positive or negative emotions that students experience in their relations with the teacher, peers, content and school (Davis et al., 2010) such as, I feel I’m in tune with the teacher, I feel that my classmates like me, etc. Social or relational engagement contributes to create a positive and healthy classroom climate depend on the quality of interactions maintained between students and the teacher, and also between peers, during the course to a great extent. Former research works in the literature on motivation provide key notions and aspects of relational engagement, such as autonomy support (Jang et al., 2010) or school belonging (Goodenow, 1993; Ros, 2014).

### Stage IV: Academic Achievement and Course Satisfaction

Learning outcomes, specifically student achievement and course satisfaction, are two of the most important indicators of a successful T-L process. “Student satisfaction is both an outcome of the learning process and a requirement for successful learning” (Sinclaire, 2014, p. 2). Accordingly, in this stage, learning outcomes and course satisfaction should be considered and evaluated. The aim of this evaluation centered on the product is, first, to know to what extent the learning objective has been achieved at the end of the course and, second, to know the level of student satisfaction reported about the followed T-L process. Student satisfaction is related to the emotions experienced by students during the course. This evaluation provides the teacher with valuable information and feedback. It allows the teacher to reflect retrospectively to introduce instructional changes for subsequent courses in order to correct failures and, thus, improve students’ achievement and course satisfaction. These changes will focus mainly on those variables that are the teacher’s responsibility; that is, learning demands and teacher support from components 1A and 1B.

## Applying Mocse to Improve Learning Outcomes

### Actions Centered on the Classroom Level

When students’ low achievement is detected in a specific subject matter at any level of education, the teacher is encouraged to use the MOCSE model to improve students’ engagement and academic results. So, based on MOCSE postulates, we suggest following a procedure that comprises two phases. Implementing the first phase (Intervention Phase 1) is recommended at the beginning of the course, a few days after the course begins, to diagnose students’ initial motivational profiles based on intention to learn indicators. To address this diagnosis, the Intention to Learn Questionnaire is provided in Annex 1.

The specific actions to be implemented into Phase 1 are listed below:

First action (Action 1): to assess “intention to learn” constructs (Expectancy-value beliefs and motivational goals) for a *diagnosis evaluation*. It should be carried out at the beginning of the course, some days after the course begins. Intention to learn is the cornerstone of the model on which the remaining components pivot: antecedents (Components 1A and 1B) and consequents (Components 3 and 4). Based on this structure, the first step consists in assessing intention to lean. “This action will provide teachers with valuable information about the extent to which students will engage in studying and working on a specific subject” (Doménech-Betoret, 2018).

Second action (Action 2): *analysis of the results* to detect the strengths and weaknesses of students’ initial motivational profiles based on intention to learn indicators (Expectancy-value beliefs and motivational goals).

Third action (Action 3): *reflection* on students’ initial motivational profile at the beginning of the course, and on the actions than I can take as a teacher. Do I continue with the planned schedule or must I introduce changes?

If appropriate, the teacher can continue with his/her established subject planning without making any changes. If the teacher notes any major deficiencies in intention to learn (either at the class or the individual level, only in some students), then a second intervention will be necessary as soon as possible (Intervention Phase 2). This second intervention aims to assess the predictive variables (demands and supports) to detect the causes responsible for students’ low motivational level. With this information, from a scientific basis, the teacher is able to initiate improvement actions by introducing the necessary changes, to avoid or reduce students’ risk of failure.

The specific actions to be implemented in Phase 2 are explained below:

Fourth action (Action 4). If deficiencies are detected, a fourth action must be implemented. This consists in designing an *Action Plan* to introduce corrective measures that aim to correct the motivational deficiencies found. It will be necessary to specify in the action plan when the improvement actions will be carried out, how, and in what way its efficacy will be evaluated. Note that the aim of the action plan is to activate and increase intention to learn (the model’s Component 3). To achieve this, it is necessary to assess the predictive or antecedent variables, and identify the specific variables responsible for students’ intention to learn according to students’ point of view; related to learning demands and supports from Components 1A and 1B. To address this assessment, the Demands and Teacher Support Questionnaire is provided in Annex 2. However, in order to collect more complete and detailed information, on the same dimensions, from students, it is recommendable to use the interview technique as a complementary methodology.

Subsequently, the instructional actions and programs that center on the aforementioned variables, which are the teacher’s responsibility and the teacher control, should be implemented. “Besides correcting motivational deficiencies, a diagnosis evaluation also allows the teacher to adjust teacher support to students’ characteristics at the beginning of the course” (Doménech-Betoret, 2018). In short, in order to activate intention to learn, appealing and meaningful demands should be planned, and affective and instructional supports (from teachers, peers, and families) should be provided.”

A decline in students’ motivation to learn (and as a result learning outcomes) has been found in the transition from Primary to Secondary Education, a period that coincides with the first years of adolescence, a difficult stage in a child’s development. Accordingly, it is especially important to use this tool at this level of education to improve students’ intention to learn and learning outcomes (academic achievement and course satisfaction). By assuming that a standard Secondary Education course comprises three trimesters and that students’ progress is evaluated and reported to parents at the end of each trimester, there are basically three time points at which the students involved in the T-L process re-update their perceptions, and almost simultaneously make decisions about what their own role and involvement must be during the course. The most important time point to check students’ intention to learn is the period when the course begins (after some days of class), but the time points corresponding “to the start of the second and third trimesters are also key due to the results obtained at the end of each trimester, provided on a report card” (Doménech-Betoret, 2018). So if we wish to check the evolution of students’ intention to learn (and perception) throughout the course, it is advisable to make evaluations at the three aforementioned time points. Psychologist, can assist teachers to implement the aforementioned actions.

### Actions Centered on the School Level

When students’ low achievement is detected in a specific school, the same actions and procedure can be followed at the school level. Previously, teachers should be trained to first understand the conceptual configuration and postulates of MOCSE, and second how it can be applied in class. The “empirical data obtained with MOCSE procedures can provide the scientific basis to design effective programs for different levels and subjects” (Doménech-Betoret, 2018), to active students’ intention to learn and, in turn, to learning outcomes like academic achievement and satisfaction at school. Finally, we wish to point out that teacher training is a fundamental element for teachers to implement changes and improvements into class. In the MOCSE context, teacher training would take two main directions; one, to train them so they are able to formulate stimulating learning demands that match the needs and interests of students in today’s society; two, so they are capable of offering students the supports they need at all times while learning.

## Discussion

In accordance with the aforementioned rationale, and based on previous research, we assert that the MOCSE model explains coherently how an educational setting operates, and provides a scientific and useful framework to be used by both researchers and teachers. On the one hand, it can be used by researchers to guide their research conducted on educational settings to explain and predict academic achievement and course satisfaction from a new approach. On the other hand, it can be used by teachers as a methodological procedure to diagnose and intervene in the classroom to improve students’ intention to learn and, consequently, learning outcomes.

The model can be applied at any level of education, to improve students’ engagement and academic results. The data obtained to date seem to indicate the model’s viability given its capacity to explain students’ engagement and academic achievement in undergraduate students (Doménech-Betoret, 2006; Doménech-Betoret et al., 2019) and Secondary Education (Abellán-Roselló, 2016). However, further research is needed at different levels of education and cultural contexts to obtain more reliable findings. After providing the model’s validity, we wish to use this tool to detect regularities in similar educational contexts, that is, at the same level of education and/or in specific curricular contents or degrees. This is a future challenge, for instance, questions such as: What are the best specific teacher supports for primary, secondary or undergraduate students? What are the best specific teacher supports for secondary students in specific curricular subjects? What is the predictive role of learning demands in primary, secondary or undergraduate students? etc. These questions and others like them are still unsolved, but are important to implement efficient instructional actions and programs to improve students’ engagement and academic achievement at a specific level or in a given curricular content.

## Author Contributions

FD-B wrote the manuscript. AG-A and LA-R reviewed the whole manuscript, checked the references, and made significant contributions. All the authors reread the manuscript and approved its submitted version.

## Conflict of Interest Statement

The authors declare that the research was conducted in the absence of any commercial or financial relationships that could be construed as a potential conflict of interest.
