# Supplementary material for: The Educational Situation Quality Model: A New Tool to Explain and Improve Academic Achievement and Course Satisfaction
Source: Front Psychol. 2019 Jul 18;10:1692. doi: 10.3389/fpsyg.2019.01692 (PMC6657589; doi:10.3389/fpsyg.2019.01692)
Supplement: Supplementary file 1 [file Data_Sheet_1.docx]

Annex-1

**MOCSE: The Intention to Learn Questionnaire**

Authors: Fernando Doménech-Betoret, Amparo Gómez-Artiga, Laura Abellán-Roselló, Adela Descals-Tomás, Esperanza Rocabert-Beút

NB: This questionnaire is the result of revising and improving that used by Doménech et al. (2019) in the Higher Education context. It would have to be adapted before being applied to other levels of education.

Dimension I: Expectancy-value beliefs

**1. Subject value**

What values does this subject have for you?

Response scale: Very little (1) – Very high (6)

1. How important is this subject for you? (Importance)

2. How useful is this subject for you? (Utility)

3. How interesting is this subject for you? (Interestingness)

4. How much time and effort are you willing to spend on this subject? (Cost)

**2. Expectation for success (Outcome expectancy + Efficacy expectation)**

Will you be successful in this subject?

Response scale: Completely disagree (1)- Completely agree (6)

1. I think that it will be easy for me to pass this subject.

2. I think that I have good aptitudes to overcome the challenges and difficulties this subject poses for me.

3. I think that I will be able to obtain good results in this subject.

4. I think that I will efficiently study and work this subject.

5. I think that I will obtain a better mark than most of my classmates.

6. I think that I have good aptitudes to properly master this subject.

7. I think that I have better aptitudes for this subject than most of my classmates.

8. I think that I will be able to successfully finish/perform the necessary activities and tasks to pass this subject.

9. I think that I have good aptitudes to fulfill the objectives set by the teacher for this subject.

10. I think that I will have find it less difficult to pass this subject than most of my classmates.

11. I think that I will be able to properly master this subject.

12. I think that I will obtain good marks in the subject’s exams.

**3. Process Expectancy: Enjoyment and feeling well**

How will you feel studying this subject?

Response scale: Quite unlikely (1) – Quite likely (6)

Feeling happy with contents:

1. Do you think you will feel food studying and working on this subject from now onward to the end of the course?

2. Do you think you will enjoy studying and working on this subject’s contents from now onward to the end of the course?

3. Do you think you will often feel positive emotions while studying and working on this subject from now onward to the end of the course?

4. Do you think you will feel profound pleasure and satisfaction from learning new things, that you do not know, in this subject?

5. Do you think you will feel profound pleasure and satisfaction as you progress and improve in this knowledge area?

Feeling happy with the teacher:

6. Do you think you will feel good at class with this teacher from now onward to the end of the course?

7. Do you think you will enjoy interacting with and learning with this teacher in class from now onward to the end of the course?

8. Do you think that, apart from learning, you will enjoy the classes given by this teacher from now onward to the end of the course?

9. Do you think you will frequently feel positive emotions with this teacher from now onward to the end of the course?

10. Do you think you will live interesting experiences in the classes given by this teacher from now onward to the end of the course?

Feeling happy with classmates:

11. Do you think you will feel good when interacting with your classmates from now onward to the end of the course?

12. Do you think you will enjoy interacting with your classmates from now onward to the end of the course?

13. Do you think you will frequently feel positive emotions when interacting with your classmates from now onward to the end of the course?

**4. Control expectations**

To what extent does it depend on you to pass or fail this subject?

Response scale: Less extent (1) – Great extent (6)

Controllability:

1. To what extent do you think your study capacity will influence your final mark for this subject?

2. To what extent do you think the effort you make in this subject will influence your final mark?

3. To what extent do you think your dedication to this subject will influence your final mark?

4. To what extent do you think your way of planning and being organized will influence your final mark?

Uncontrollability**:**

5. To what extent do you think you getting on well with the teacher will influence your final mark?

6. To what extent do you think your teacher making exams complicated will influence your final mark?

7. To what extent do you think you being lucky in exams will influence your final mark?

8. To what extent do you think the type of questions the teacher presents in exams will influence your final mark?

9. To what extent do you think the teacher’s degree of subjectivity will influence your final mark?

10. To what extent do you think that the syllabus chosen by the teacher will influence your final mark?

Dimension II: Achievement goals

What is your objective with this subject?

Response scale: Completely disagree (1)- Completely agree (6)

**1. Mastery goal***:

1. My objective with this subject is to learn all I can.

2. My objective with this subject is to learn new things.

3. My objective with this subject is to progress and acquire new knowledge.

4. My objective with this subject is to improve my competences and skills in this field.

5. My objective with this subject is to master all I can.

**2. Performance goal***:

6. My objective with this subject is to show my classmates and my teacher I am good in this subject.

7. My objective with this subject is to show my classmates and my teacher I am able to easily do this subject’s tasks and activities.

8. My objective with this subject is to show my classmates and my teacher I am able to easily progress in this subject.

9. My objective with this subject is to show my classmates and my teacher I am intelligent.

10. My objective with this subject is to show my classmates and my teacher I am able to master this subject.

**3. Performance-avoidance goal*****:**

11. My objective with this subject is to avoid my classmates and my teacher thinking I´m silly.

12. My objective with this subject is to my classmates and my teacher thinking I’m ignorant.

13. My objective with this subject is to avoid my teacher thinking I´m not as clever as my classmates.

14. My objective with this subject is to avoid making a fool of myself when the teachers asks questions or asks me go to the blackboard.

15. My objective with this subject is to avoid the teacher thinking I have problems mastering this subject.

**4. Self-worth goal**:

16. My objective with this subject is to experience the pride felt after being successful.

17. My objective with this subject is to experience the satisfaction of overcoming the difficulties and challenges I may face.

18. My objective with this subject is to feeling self-satisfied with work well done.

**5. Avoidance goal**:

19. My objective with this subject is to pass it by making the least effort.

20. My objective with this subject is to not making more effort than strictly necessary.

21. My objective with this subject is seeking shortcuts to pass the subject by making the least effort.

22. My objective with this subject is to studying and working just enough to pass.

* Adapted from: Midley, C. et al. (2000). *Manual for the Patterns of Adaptive Learning Scales*. University of Michigan.

Annex-2

**MOCSE: The Learning Demands and Teacher Support Questionnaire.**

**(Controllable variables)**

Authors: Fernando Doménech-Betoret, Amparo Gómez-Artiga, Laura Abellán-Roselló, Adela Descals-Tomás, Esperanza Rocabert-Beút

NB 1: This questionnaire is the result of revising and improving that used by Doménech et al. (2019) in the Higher Education context. It would have to be adapted before being applied to other levels of education

NB 2: The same dimensions should be addressed using a more qualitative technique, such as an interview, and complement the information collected with the questionnaire.

Dimension I: LEARNING DEMANDS

Response scale: Completely disagree (1)- Completely agree (6)

**Difficulty of demands**

1. The objectives set in this subject to be fulfilled are too demanding.

2. The workload I am expected to do to pass this subject is too much.

3. It takes too much effort to obtain a good mark in this subject.

4. The expected level of demand to pass the theoretical part of this subject is too high.

5. The expected level of demand to pass the practical part of this subject is too high.

6. The evaluation criteria set by the teacher to pass this subject are too demanding.

**The intrinsic value of demands**

1. The activities and tasks I am asked to do during this course to pass the subject are appealing.

2. The activities and tasks I am asked to do during this course to pass the subject are innovative.

3. The activities and tasks I am asked to do during this course to pass the subject match my current interests and preferences.

4. The activities and tasks I am asked to do during this course to pass the subject match today’s reality.

5. The activities and tasks I am asked to do during this course to pass the subject match my personal and/or professional requirements.

6. The activities and tasks I am asked to do during this course have aroused my curiosity.

7. The contents I will have to study in this subject are appealing.

8. The contents I will have to study are interesting.

9. The contents I will have to study have aroused my curiosity.

10. The contents I will have to study have aroused my interest.

Dimension II: TEACHER SUPPORT

Response scale: Completely disagree (1)- Completely agree (6)

**1. Content comprehension support**

1. The teacher's explanations are clear and understandable.

2. The teacher's explanations are easy to follow.

3. The teacher's explanations match the students’ level to understand them.

4. The teacher's explanations are logical and well-organized.

**2. Motivational support**

1. The teacher has transmitted his/her interest and enthusiasm right from the start.

2. The teacher has made an effort to arouse our curiosity about and interest in this subject right from the start.

3. From what I´ve seen on the first days of class, I think that the teacher’s explanations will be engaging and entertaining.

4. From what I´ve seen on the first days of class, I think that the teacher will make the effort for students to learn and enjoy learning.

5. From what I´ve seen on the first days of class, I think that the teacher will make the effort to avoid students feeling bored in class.

6. From what I´ve seen on the first days of class, I think that the teacher will be able to maintain students’ attention and interest.

**3. Formative evaluation**

1. From the way the evaluation is set out, I think that the teacher will supervise my work to correct mistakes before works are handed in.

2. From the way the evaluation is set out, I think that it will help us to keep up-to-date with the subject and that efforts will be shared throughout the course.

3. The evaluation system attaches much importance to students’ continuous work and to the teacher’s feedback.

4. To decide on a final mark, the teacher will bear in mind students’ efforts and continued progress throughout the course (supervising tasks, preparing tests, etc.)

**4. Teacher-student relational support**

1. The teacher makes efforts to understand us.

2. The teacher comes over as being close.

3. The teacher comes over as being willing and open to dialog.

4. The teacher treats us with respect.

5. The teacher shows interest in our problems.

**5. Competence support**

1. The teacher has transmitted right from the start the idea that we are all able to pass this subject if we want to.

2. The teacher has reassured us right from the start by saying that this subject is not hard to pass.

3. The teacher has made us feel good right from the start by stating that being successful in this subject depends on us.

4. The teacher has made us feel competent right from the start to master this subject.

5. The teacher has transmitted right from the start the idea that we can progress and obtain good results in this subject.

**6. Recognition support (implication and effort)**

1. The teacher appreciates and recognizes us when we do things well.

2. The teacher appreciates and recognizes us when we make efforts.

3. The teacher appreciates and recognizes us when we follow his/her guidance and guidelines.

4. The teacher appreciates and recognizes us when we actively engage in learning.

5. The teacher appreciates and recognizes us when we work beyond what is expected of us.

**7. Study guidance**

1. The teacher offers us guidance about how to learn more and better in this subject.

2. The teacher offers us guidance about how to perform more in this subject.

3. The teacher offers us guidance about how to be successful in this subject.

4. The teacher offers us guidance about how to face learning this subject.

5. The teacher offers us guidance about how to be organized and to plan to obtain good results in this subject.

**8. Autonomy support**

1. The teacher offers us the chance to focus on and organize work on themes as we wish.

2. The teacher offers us the chance to choose from a wide range of tasks, activities, readings, etc., depending on our preferences.

3. The teacher offers us the chance to focus on and organize the way we perform tasks as we wish.

4. Although the teacher respects the subject’s syllabus, (s)he encourages us to make our own decisions about how to focus on work and study the subject.

5. The teacher encourages us to make self-assessments of our learning to be aware of our successes and mistakes.

6. The teacher encourages us feel free to express our ideas and opinions.

7. The teacher encourages us to shoulder our responsibilities in all the tasks that depend on us (perform the required tasks, meet deadlines, listen to explanations, etc.).

**9. Providing didactic resources to study support**

1. The teacher has provided us with enough varied materials to study and work on this subject.

2. The materials provided by the teacher to study and work on this subject are clear and comprehensive.

3. The materials provided by the teacher to study and work on this subject are accessible and easy to find.

4. The materials provided by the teacher to study and work on this subject are of good quality.

**10. Teacher’s accessibility (willingness)**

1. The teacher is always willing to solve students’ doubts.

2. The teacher worries about our learning and is always willing to help.

3. The teacher is always willing to guide us when we encounter difficulties completing a task.

4. The teacher quickly and efficiently solves students’ doubts.

5. The teacher is always accessible either in person or from a distance.

FINAL NOTE: Researchers and teachers can access future updates of the questionnaires at the following website: <https://www3.uji.es/~betoret/index.html> (MOCSE section).
